# Stimulus Variability Affects the Amplitude of the Auditory Steady-State Response

**DOI:** 10.1371/journal.pone.0034668

**Published:** 2012-04-03

**Authors:** Michael I. G. Simpson, William P. Woods, Garreth Prendergast, Sam R. Johnson, Gary G. R. Green

**Affiliations:** 1 York Neuroimaging Centre, University of York, York, United Kingdom; 2 Swinburne University of Technology, Melbourne, Australia; Max Planck Institute for Human Cognitive and Brain Sciences, Germany

## Abstract

In this study we investigate whether stimulus variability affects the auditory steady-state response (ASSR). We present cosinusoidal AM pulses as stimuli where we are able to manipulate waveform shape independently of the fixed repetition rate of 4 Hz. We either present sounds in which the waveform shape, the pulse-width, is fixed throughout the presentation or where it varies pseudo-randomly. Importantly, the average spectra of all the fixed-width AM stimuli are equal to the spectra of the mixed-width AM. Our null hypothesis is that the average ASSR to the fixed-width AM will not be significantly different from the ASSR to the mixed-width AM. In a region of interest beamformer analysis of MEG data, we compare the 4 Hz component of the ASSR to the mixed-width AM with the 4 Hz component of the ASSR to the pooled fixed-width AM. We find that at the group level, there is a significantly greater response to the variable mixed-width AM at the medial boundary of the Middle and Superior Temporal Gyri. Hence, we find that adding variability into AM stimuli increases the amplitude of the ASSR. This observation is important, as it provides evidence that analysis of the modulation waveform shape is an integral part of AM processing. Therefore, standard steady-state studies in audition, using sinusoidal AM, may not be sensitive to a key feature of acoustic processing.

## Introduction

The auditory steady-state response (ASSR) is a clinically robust tool [1]–[3], which is used to study the dynamics of cortical following responses to sinusoidally amplitude modulated stimuli, and may be recorded with both EEG [4]–[6] and MEG [7]–[9]. Although the ASSR is known to be highly reliable, the order of stimulus presentation can affect amplitude modulation (AM) detection thresholds. Behavioural studies have shown that pre-exposure to AM affects AM detection thresholds, with both sinusoidal and non-sinusoidal adapting AM stimuli [10]–[13], and also that the degree of adaptation is dependent on the waveform shape [11]. Neurophysiologically, AM adaptation has also been shown to affect neural firing rates in the auditory cortex of marmoset monkeys [14].

Time-reversing asymmetric triangular AM, to generate ‘ramped’ and ‘damped’ AM, results in stimuli that have different behavioural detection thresholds but identical modulation spectra [15]–[16]. The discrimination of ramped AM is dependent on the slope of the onset ramp, relative to the modulation cycle [17]; indicating that modulation processing is dependent on waveform shape, rather than the modulation spectrum. A comparable finding was observed by Prendergast *et al.* [18] using MEG to study the ASSR to different widths of cosinusoidal pulsed AM stimuli, who show that the magnitude of the ASSR is dependent on the waveform shape rather than the modulation spectra, and is selective for the most prevalent waveform shapes in speech [19].

In this MEG study we use raised cosinusoidal pulsed AM stimuli, used by Prendergast *et al.* [18]. A key property of these stimuli is that they allow manipulation of the modulation waveform shape, independent of the modulation rate. We use these stimuli to explore whether stimulus variability affects the amplitude of the ASSR. We use three different pulse widths of cosinusoidal AM, and present them as stimuli which either have a repetitive waveform shape, or a waveform that varies pseudo-randomly between pulse widths, to test whether variability in the waveform shape affects the amplitude of the ASSR.

## Methods

### Participants and Ethics Statement

Data were recorded from 21 participants. All participants had no known hearing disorders. Participants provided written informed consent. The study was approved by the ethics committee of the York Neuroimaging Centre, and was in accordance with the Declaration of Helsinki. One participant was removed from the study due to an anomaly on their MRI scan, and two further participants were removed due to moving too much during data acquisition. The 18 participants (11 female, 7 male) whose data were analysed had a mean age of 22.3 years, with a standard deviation of 3.1 years.

### Stimuli

**Figure 1:**
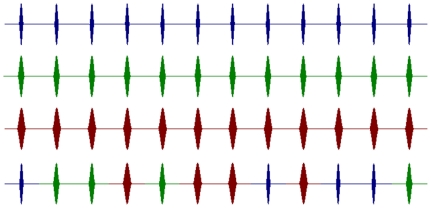
A schematic of the four pulsed AM stimuli. Stimuli are 3 s long, with a modulation rate of 4 Hz. Blue pulses have a 16 ms half-width, maroon pulses a 24 ms half-width, and green pulses a 32 ms half-width. The top three waveforms we refer to as the fixed-width modulations, and the bottom waveform, with a combination of pulse widths, is referred to as a mixed-width modulation. The pseudo-random order of the mixed-width stimuli is; 16 ms; 24 ms; 24 ms; 32 ms; 24 ms; 32 ms; 32 ms; 16 ms; 32 ms; 16 ms; 16 ms; 24 ms. All stimuli are modulations of a 500 Hz sinusoid carrier waveform.

The stimuli used in this study were specifically chosen to evoke a strong ASSR. We use the three widths of raised cosinusoidal pulsed AM from Prendergast *et al.* [18] that gave the greatest average responses; these were cosinusoidal AM pulses with pulse half-widths of 16 ms, 24 ms and 32 ms. These pulsed AM stimuli were either presented as repetitions of the same modulation half-width (referred to as fixed-width stimuli), or as a stimulus that had a combination of the three modulation half-widths (referred to as mixed-width stimuli), see Figure 1. The design of the study has an internal control, and simply tests whether the ASSR to the mixed-width AM pulsed stimuli is significantly different to the average ASSR to the three fixed-width AM stimuli. Our null hypothesis is that there will be no significant difference between the ASSR to the mixed-width AM stimuli, and the average ASSR to the three fixed-width AM stimuli.

The cosinusoidal pulsed AM modulated a 500 Hz carrier waveform, with a modulation depth of 90%. Each AM stimulus was presented at 4 Hz, and had a duration of 3 s; hence each AM waveform contained 12 cosinusoidal pulses. The fixed-width AM stimuli had 12 repetitions of either the 16 ms, 24 ms or 32 ms modulation half-widths, the mixed-width AM had 4 of each of the 16 ms, 24 ms or 32 ms modulation half-widths, presented in a pseudo random order (see Figure 1). There were 42 repeats of each AM stimuli, plus 42 repeats of a 3 s 500 Hz pure tone, and 42 repeats of 3 s of silence. The six stimulus sets were interleaved and presented in a random order, with an inter-stimulus-interval of 1 s. Stimuli were presented monaurally to the left ear only. The whole experiment took 16 minutes and 47 seconds. Stimuli were presented via Etymotic Research ER3-A insert headphones (Etymotic Research Inc., Illinois) at 75 dB SPL.

### Acquisition

Data were collected using a Magnes 3600 whole-head 248-channel magnetometer (4-D Neuroimaging Inc., San Diego). The data were recorded with a sample rate of 678.17 Hz and low-pass filtered at 200 Hz. Prior to acquisition, five facial landmark head-coils and a digital head-shape were recorded using a Polhemus Fastrak Digitization System, which derive the landmark head-coil locations, and the digital head-shape location in relation to the position of the MEG sensors. The landmark head-coil locations were used to measure the head position in the scanner before and after acquisition. The digitised head-shape was used for coregistering the MEG data with the participants structural MRI.

### Coregistration

Participants digitised head-shapes were coregistered with a participants' T1 weighted structural MR scan using an adaptation of the technique described by Kozinska *et al.* [20]. T-1 weighted MR images were acquired with a GE 3.0 T Signa Excite HDx system (General Electric, Milwaukee, USA) using an eight-channel head coil and a 3-D fast spoiled gradient-recalled sequence: TR/TE/flip angle = 8.03 ms/3.07 ms/20°; spatial resolution of 1.13 mm×1.13 mm×1.0 mm; in-plane resolution of 256×256×176 contiguous slices.

For each participant, their structural MRI scan was skull-stripped using the BET tool in FSL [21]–[22]. We then spatially normalized the skull-stripped MRI scans to the Montreal Neurological Institute (MNI) 152 standard 1 mm brain, which is based on the average of 152 individual T-1 weighted structural MR images [23]. Spatial normalisation was performed using the diffeomorphic non-linear SyN transform within ANTS [24].

### Analysis

MEG datasets were manually artefact rejected by visually inspecting trials and excluding from the analysis any trials that contained physiological or non-physiological artefacts. Across the 18 participants, 252 epochs were analysed per subject, and a mean of 15.1 epochs *(s. dev. = 7.5 epochs)* were rejected.

A group analysis was performed in source-space using beamformer inverse modelling. A uniform 5 mm grid was generated on the MNI brain, and for each individual this grid was transformed to an irregular grid on their individual T1 structural MRI using the inverse of their nonlinear SyN transform. The data were inverse modelled using a vectorized, linearly constrained minimum-variance (LCMV) beamformer [25], modified as referenced in Huang *et al.* [26] as a Type I beamformer. To measure the 4 Hz ASSR at each location in source space, we averaged across the trials for each stimulus condition and measured the amplitude of the 4 Hz component of the FFT in each of the x, y and z directions, and then summed these to get the total activity at that location.

To generate mean and variance estimates for the FFT calculations across all trials, we used jackknife re-sampling [27]–[28]. To enable us to compare the mean 4 Hz component of the three fixed-width ASSRs with the 4 Hz component of the mixed-width ASSR, we pooled the mean and variance jackknife statistics across the three fixed-width conditions. Pooling of the jackknife mean *(* *eq. 1* *)* and standard deviation *(* *eq. 2* *)* across the three fixed-width conditions was done using the following formula:
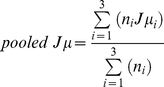
(1)

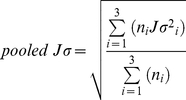
(2)Where *Jμ* is the jackknife mean, *Jσ* the jackknife standard deviation, *Jσ^2^* the jackknife variance, *_i_* is the condition (*fixed-width 16 ms, fixed-width 24 ms, fixed-width 32 ms*) and *n* is the number of jackknife re-samples for that condition, determined by the number of clean epochs.

For group level analysis, the pooled mean 4 Hz component for the fixed-width ASSRs was compared with the mean 4 Hz component for the mixed-width ASSR, using a non-parametric permuted unpaired t-test [29]. These group statistics were performed on one region of interest (ROI) in the right hemisphere. In the defined ROI, maximum statistics on voxel values (single threshold as opposed to cluster size) were used to correct for the Family-Wise Error in individuals [29].

**Figure 2:**
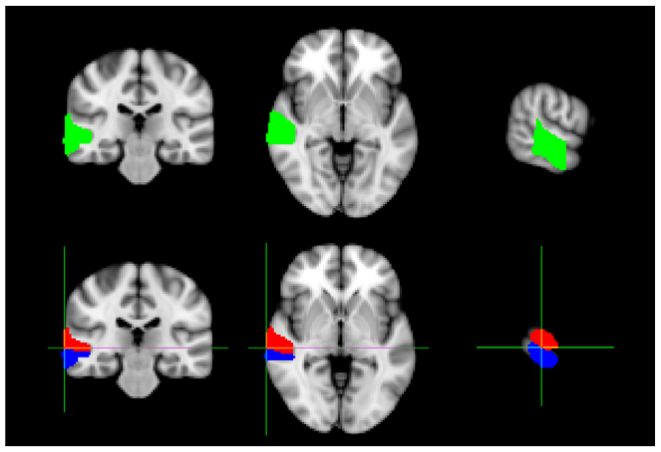
Beamforming ROI. The green ROI in the upper images includes the posterior divisions of both the middle and superior temporal gyri. This is the ROI that was used for the beamforming contrasts, and was generated by selecting the right hemisphere section of areas 10 and 12 in the Harvard Oxford atlas. In the lower image is the peak voxel from the Prendergast 2010 study; MNI 70, −26, −2, with the right hemisphere sections of areas 10 (red) and 12 (blue).

The ROI was based upon the location of the most consistent response to a variety of cosinusoidal pulsed AM, and a sinusoidal AM, in Prendergast *et al.* [18], which was centred at the MNI co-ordinate 70, −26, −2. This location was used as a seed point to choose a specific ROI from the Harvard-Oxford cortical atlas. The seed MNI co-ordinate was located on the border between the posterior divisions of the Middle and Superior Temporal Gyri (MTG/STG), in the right hemisphere. Hence, an ROI was defined that included the posterior divisions of the both the middle and superior temporal gyri, by selecting the right hemisphere section of areas 10 and 12 in the Harvard Oxford atlas (see Figure 2).

To confirm the suitability of this area as an ROI in this study, we perform two analyses. Firstly, using a virtual electrode at the MNI co-ordinate 70, −26, −2, we calculate the average spectra of the ASSR to each of the four AM stimuli. We sum the spectra across the x, y and z directions, and average these across the 18 participants. These four spectra are then normalised by the amplitude of the 4 Hz component in the response to the mixed-width stimuli. We also plot the 4 Hz component of each of the four ASSRs against the 4 Hz component of the respective stimulus waveforms. The energy in the stimulus waveforms we normalised by the amplitude of the 4 Hz component in the mixed-width stimuli. These initial analyses are principally performed to confirm the presence of a robust 4 Hz response at the MNI co-ordinate 70, −26, −2. The virtual electrodes were generated using a vectorized, linearly constrained minimum-variance (LCMV) beamformer [25], [30]. We identified the MNI coordinate 70, −26, −2 in the non-linearly transformed brain in each participant, and then this location was re-warped back using the inverse SyN transform within ANTS, back to the individual's structural MRI. Virtual electrodes were generated from the re-warped, inverse transformed beamforming grid, and were unfiltered. As a secondary confirmation of suitability we also performed group level beamforming analyses following the beamforming methods outlined previously, and compare the mean 4 Hz component in the ASSR to each of the four AM conditions, to the 4 Hz component in the response to the unmodulated 500 Hz pure tone. This secondary analysis is to confirm that a strong 4 Hz response is observable with the spectral amplitude measure we use in our experimental contrast. It also allows us to compare the sources from this amplitude based metric, with the amplitude and phase based T2 metric used by Prendergast *et al.* [18].

As a final analysis we use the same beamforming methods to contrast the 4 Hz component in ASSR to the mixed-width stimuli, with the 4 Hz component in ASSRs to each of the three fixed-width stimuli. This allows us to compare the mixed-width responses with the individual fixed-width responses, rather than with the pooled fixed-width responses as is done in the main experimental contrast.

## Results

### Verification of ROI selection

#### Virtual Electrode Analysis

**Figure 3:**
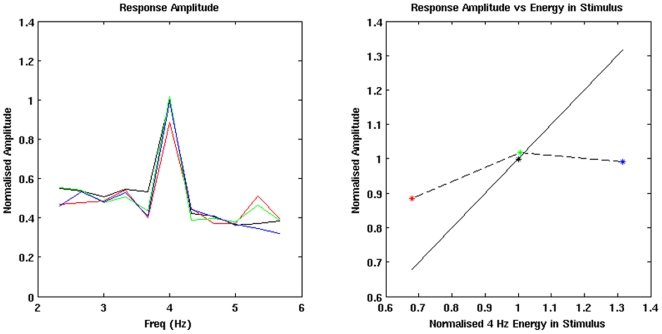
Virtual electrode analysis of the ROI. Normalised response amplitudes for the four AM conditions. For the lines in the left plot, and the points in the right plot, the following colours are used; mixed-width (black); fixed-width 16 ms (red); fixed-width 24 ms (green); fixed-width 32 ms (blue). The amplitude of the FFT spectra are normalised by the amplitude of the 4 Hz component in the ASSR to the mixed-width stimuli, the 4 Hz energy in the stimulus waveforms are normalised by the 4 Hz energy in the mixed-width waveform. The normalised energy at 4 Hz in the four stimulus waveforms are; fixed-width 16 ms, 0.68; fixed-width 24 ms, 1.01; fixed-width 32 ms, 1.32; mixed-width, 1. The normalised amplitude at 4 Hz in the FFT spectra of the four responses are; fixed-width 16 ms, 0.88; fixed-width 24 ms, 1.02; fixed-width 32 ms, 0.99; mixed-width, 1. When the response amplitude is plotted against the energy in the waveform, right plot, the solid black line shows what would be predicted if there was a linear relationship between the 4 Hz component in the ASSR and the stimulus waveform. The dashed black line is the observed relationship for the three fixed-width AM conditions.

To confirm that we observed a clear 4 Hz following response at the location of the most consistent following response in Prendergast *et al.* [18], MNI co-ordinate 70, −26, −2, we calculate the grouped average spectra in the responses to each of the four AM stimuli. The spectra are then normalised by the amplitude of the 4 Hz component in the response to the mixed-width AM stimuli, see Figure 3 (left plot). We also plot the normalised 4 Hz components of the four ASSRs against the normalised 4 Hz components of the stimulus waveforms, see Figure 3 (right plot). In these plots of the average virtual electrode spectra we observe a distinct peak at 4 Hz, indicating that there is a strong 4 Hz ASSR for each condition, which is present across the group of participants. The normalised amplitudes of the 4 Hz components in the group-averaged ASSRs are; fixed-width 16 ms, 0.88; fixed-width 24 ms, 1.02; fixed-width 32 ms, 0.99; mixed-width, 1. The mean normalised amplitude across the fixed-width presentations is 0.96 of the amplitude of the mixed width response. Based on the statistics in the beamforming contrasts between the mixed-width and each of the fixed-width responses, which are presented later in the Results section, only the 4 Hz component for the fixed-width 16 ms ASSR is significantly different from the 4 Hz component for the mixed-width ASSR, at this location.

#### Group Analysis

**Figure 4:**
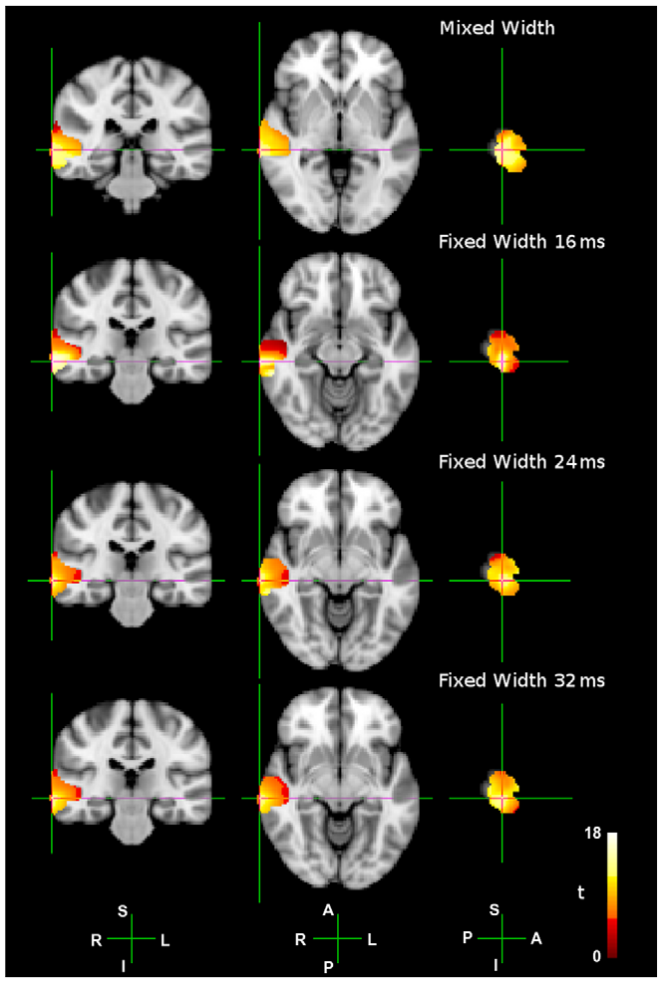
Group level t-tests between the 4 Hz AM responses and the pure tone response. Group level unpaired t-tests between the 4 Hz component of the ASSRs to each of the four AM conditions; mixed-width; fixed-width 16 ms; fixed-width 24 ms; fixed-width 32 ms, and the 4 Hz component of the response to a 500 Hz pure tone. A hot colour scheme is used in each figure, showing activity between the respective p = 0.05 cut-off threshold (see Table 1), and a max t-value of *t = 18.0*. For peak locations, refer to Table 1. Anatomical axis are labelled as follows; R, right; L, left; A, anterior; P, posterior; S, superior; I, inferior.

We compared the 4 Hz component of the ASSR to each of the four AM conditions, with the 4 Hz component of the response to the unmodulated 500 Hz pure tone, using an unpaired non-parametric permuted t-test [29]. Statistical thresholds were determined using maximum statistics on voxel values [29]. These t-maps are shown in Figure 4, and peak locations and max t-values are in Table 1. For each of the four AM conditions, when we contrast the 4 Hz components in the respective ASSRs to the 4 Hz component in the response to the pure tone, we see highly significant peaks of activity within the ROI. The p = 0.05 values range between *t = 2.52* to *t = 2.75*, across the four AM conditions, and the max t-values range between *t = 14.70* and *t = 17.16* (see Table 1). Therefore, there are clear and statistically significant ASSRs to each of the AM stimuli. The location of the peaks in all four AM conditions; mixed-width, MNI coordinate (*70, −32, −2*); fixed-width 16 ms, MNI coordinate (*70, −26, −12*); fixed-width 24 ms, MNI coordinate (*70, −26, −8*); fixed-width 32 ms, MNI coordinate (*70, −26, −8*); are in close proximity to the seed location from Prendergast *et al.* [18], MNI coordinate *(70, −26, 2)*. Note, the location of the mixed-width peak is slightly posterior to the location of the three fixed-width peaks.

**Table 1:** Max t-values and p = 0.05 threshold t-values, for each MNI peak co-ordinate taken from the contrasts of the 4 Hz components of the ASSRs to each of the four AM conditions; mixed-width; fixed-width 16 ms; fixed-width 24 ms; fixed-width 32 ms, and the 4 Hz component of the response to a 500 Hz pure tone, plotted in Figure 4.

	MNI co-ordinate	t-value (p = 0.05)	t-value (max)
**Mixed-width**	70, −32, −2	2.52	15.16
**Fixed-width 16 ms**	70, −26, −12	2.75	17.16
**Fixed-width 24 ms**	70, −26, −8	2.72	14.70
**Fixed-width 32 ms**	70, −26, −8	2.55	14.89

### Analysis of Mixed-width vs Pooled Fixed-width responses

#### Individual z-maps

**Figure 5:**
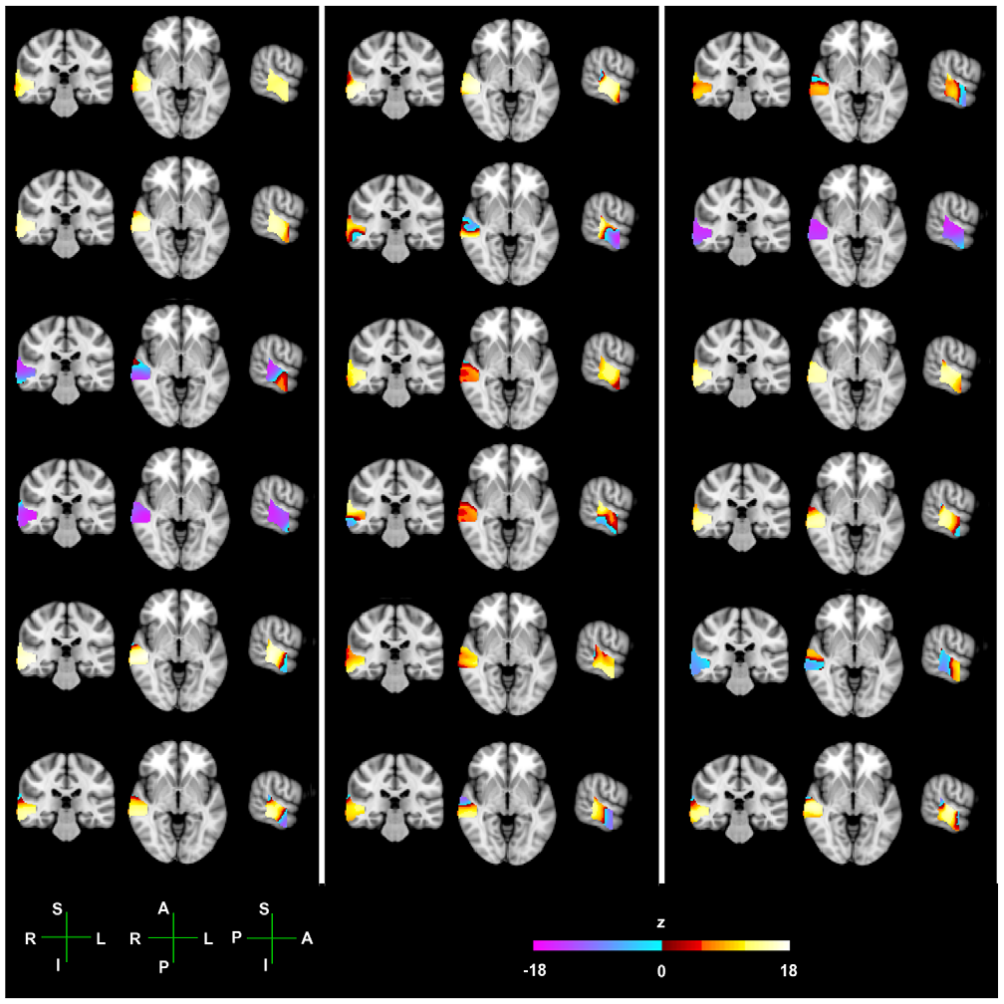
Individual level z-values for the contrast between the mixed width and fixed width responses. Individual level z-maps, showing the first-level statistics of the contrast between the 4 Hz component of the mixed-width ASSRs, and the pooled fixed-width ASSR. The hot colour scheme shows positive z-values, where the mixed-width ASSR is greater than the fixed-width ASSR. The cool colour scheme shows negative z-values, where the fixed-width ASSR is greater than the mixed-width ASSR. Activity is plotted between the respective p = 0.05 threshold (see Table 1), and a max z-value of *z = 18.0*. For peak locations, within the defined ROI, refer to Table 1. Anatomical axis are labelled as follows; R, right; L, left; A, anterior; P, posterior; S, superior; I, inferior.

At the individual level, before we perform the group level analysis, the 4 Hz component from the pooled fixed-width ASSR are contrasted with the 4 Hz component from the mixed-width ASSR, and plotted as z-maps for each participant (see Figure 5). These individual z-maps for each participant show where the 4 Hz component in the mixed-width ASSR is greater than the 4 Hz component for the pooled fixed-width ASSR (positive z-values, plotted in a hot colour scheme); and where the 4 Hz component in the pooled fixed-width ASSR is greater that the 4 Hz component from the mixed-width ASSR (negative z-values, plotted in a cool colour scheme). The MNI co-ordinates of the peak locations for when the mixed-width ASSR is greater, max values, and when the pooled fixed-width ASSR is greater, min values, are in Table 2. In 16 of the 18 participants there are both positive and negative z-values in the ROI, and of these 16 participants, 13 have their greatest absolute z-value in the positive direction, and 3 have their greatest absolute z-value in the negative direction. The average maximum positive z-value across the 17 participants who show positive z-values is *z = 14.64*. The average minimum negative z-value across the 17 participants who show negative z-values is *z = −10.10*.

**Table 2:** Maximum and minimum MNI coordinates, with associate z-values, for a given sign of z-value.

	Max MNI Coordinate			Max positivez-value	Min MNI Coordinate			Min Negativez-value
	x	y	z		x	y	z	
**1**	46	−30	4	***16.12***	N/A	N/A	N/A	***0***
**2**	46	−26	0	***16.35***	68	−8	−18	***−0.20***
**3**	64	−16	−28	***7.91***	70	−36	8	***−15.50***
**4**	68	−20	14	***9.46***	46	−30	−2	***−15.97***
**5**	46	−26	−2	***17.74***	56	−10	−30	***−7.25***
**6**	58	−32	−12	***15.84***	60	−12	−28	***−11.77***
**7**	52	−30	−8	***17.21***	68	−36	14	***−12.09***
**8**	60	−36	2	***15.20***	60	−8	−16	***−15.79***
**9**	46	−32	−6	***14.91***	52	−32	−8	***−2.55***
**10**	64	−30	14	***13.92***	68	−32	−18	***−8.67***
**11**	56	−20	−22	***14.62***	70	−36	2	***−5.39***
**12**	60	−36	2	***14.37***	68	−10	−2	***−11.48***
**13**	66	−34	22	***14.86***	54	−10	−22	***−10.10***
**14**	N/A	N/A	N/A	***0***	56	−36	8	***−15.98***
**15**	66	−20	8	***16.84***	66	−34	22	***−6.78***
**16**	46	−26	−4	***15.51***	68	−36	18	***−10.97***
**17**	68	−8	−18	***12.00***	60	−32	−16	***−9.14***
**18**	56	−20	−2	***16.15***	68	−36	18	***−12.15***

These values are derived from the respective individual level z-maps in Figure 5. These figures show the first-level statistics of the contrast between the 4 Hz component of the mixed-width ASSRs, and the pooled fixed-width ASSR.

#### Group level t-maps

**Figure 6:**
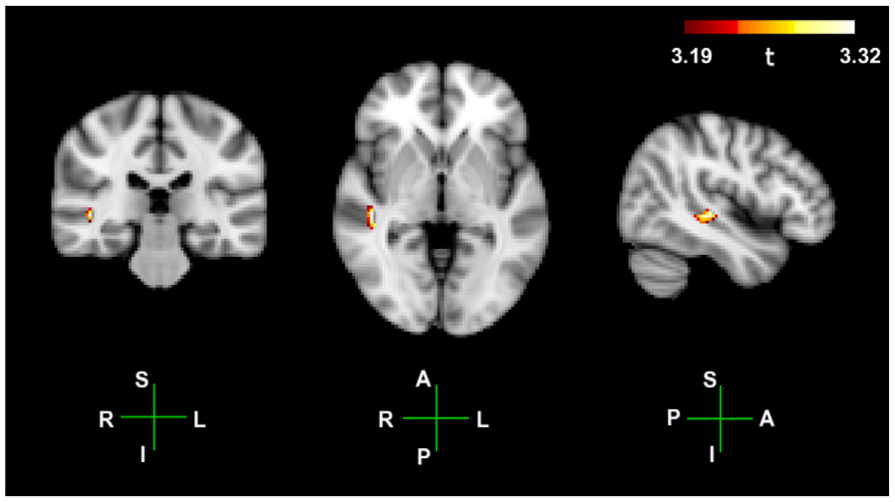
Group level t-tests between the mixed width responses and the pooled fixed width responses. Group level unpaired t-test between the 4 Hz components of the ASSRs to the mixed-width AM stimulus, and the pooled fixed-width AM stimuli. The hot colour scheme plots significant activity between the two conditions, within the ROI; p = 0.05 threshold is *t = 3.19*, max *t = 3.32*. Peak activity is observed at the MNI coordinate 46, −26, −2. Anatomical axis are labelled as follows; R, right; L, left; A, anterior; P, posterior; S, superior; I, inferior.

For the group level beamformer analysis we compared the 4 Hz component from the pooled fixed-width ASSR, with the 4 Hz component from the mixed-width ASSR, across the 18 participants using an unpaired non-parametric permuted t-test [29]. Statistical thresholds were determined using maximum statistics on voxel values [29]. A group level t-map, thresholded at p = 0.05 (*t = 3.19*), is plotted in Figure 6. In Figure 6, all the significant voxels have positive t-values; there are no significant voxels with negative t-values. The largest t-value in the negative direction is *t = −0.2*. As the sign of activity is the same as in Figure 5, this indicates that we only get a significant group level effect where the 4 Hz component in the mixed-width ASSR is greater than the 4 Hz component for the pooled fixed-width ASSR. The significant activity has a locus which peaks at the MNI coordinate 46, −26, −2, with a max t-value of *t = 3.32*. This locus of activity lies on the inferior surface of the STG, near the medial junction of the STG and MTG.

#### Virtual Electrode Analysis

**Figure 7:**
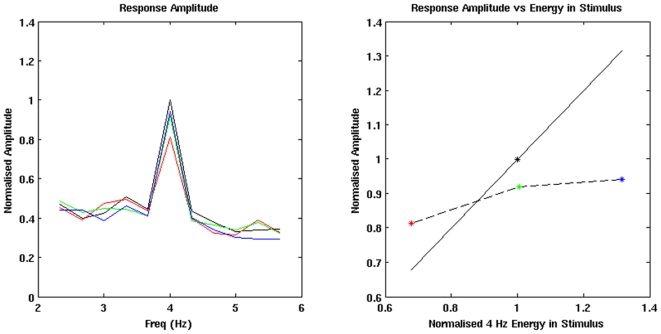
Virtual electrode analysis at the peak of the difference in the group level contrast between the mixed width and pooled fixed width responses. Normalised response amplitudes for the four AM conditions. For the lines in the left plot, and the points in the right plot, the following colours are used; mixed-width (black); fixed-width 16 ms (red); fixed-width 24 ms (green); fixed-width 32 ms (blue). The amplitude of the FFT spectra are normalised by the amplitude of the 4 Hz component in the ASSR to the mixed-width stimuli, the 4 Hz energy in the stimulus waveforms are normalised by the 4 Hz energy in the mixed-width waveform. The normalised energy at 4 Hz in the four stimulus waveforms are; fixed-width 16 ms, 0.68; fixed-width 24 ms, 1.01; fixed-width 32 ms, 1.32; mixed-width, 1. The normalised amplitude at 4 Hz in the FFT spectra of the four responses are; fixed-width 16 ms, 0.81; fixed-width 24 ms, 0.92; fixed-width 32 ms, 0.94; mixed-width, 1. When the response amplitude is plotted against the energy in the waveform, right plot, the solid black line shows what would be predicted if there was a linear relationship between the 4 Hz component in the ASSR and the stimulus waveform. The dashed black line is the observed relationship for the three fixed-width AM conditions.

To confirm that a clear following response was present at the peak of the difference in the group analysis, MNI coordinate 46, −26, −2, we calculate the grouped average spectra in virtual electrodes from the 18 participants, using the same methods that were used to generate the plots in Figure 3. In the FFT spectra for each waveform, see Figure 7, there are distinct peaks at 4 Hz, and notably the 4 Hz peak for the mixed-width ASSR is greater than the 4 Hz peak in any of the fixed-width ASSRs. This is consistent with the significant difference observed in the group level t-map. The normalised amplitudes of the 4 Hz components in the group-averaged ASSRs are; fixed-width 16 ms, 0.81; fixed-width 24 ms, 0.92; fixed-width 32 ms, 0.94; mixed-width, 1. The mean normalised amplitude across the fixed-width presentations is 0.89 of the amplitude of the mixed width response. Based on the statistics in the beamforming contrasts between the mixed-width and each of the fixed-width responses, which are presented later in the Results section, only the 4 Hz component for the fixed-width 16 ms ASSR is significantly different from the 4 Hz component for the mixed-width ASSR, at this location.

### Analysis of Mixed-width vs Individual Fixed-width responses

#### Group level t-maps

**Figure 8:**
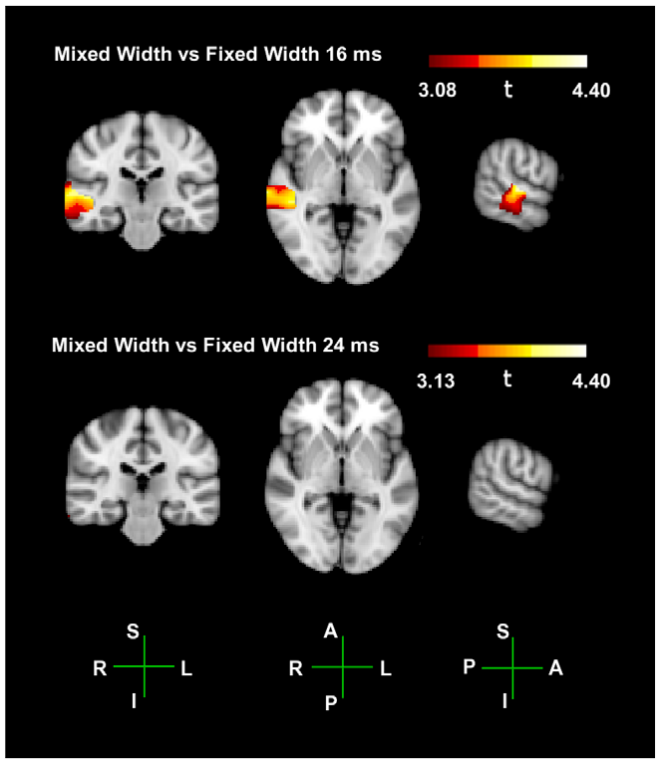
Group level t-tests between the mixed width responses and the individual fixed width responses. Group level unpaired t-test between the 4 Hz components of the ASSRs to the mixed-width AM stimulus, and the individual fixed-width AM stimuli. The hot colour scheme plots significant activity between the two conditions within the ROI, p = 0.05 significant values are used as cut-off thresholds. Results are not plotted for the fixed-width 32 ms contrast, as there were no significant voxels. Anatomical axis are labelled as follows; R, right; L, left; A, anterior; P, posterior; S, superior; I, inferior.

To further understand the relationship between the mixed-width responses and each individual fixed-width response, we use the same group-level beamformer contrasts to compare the 4 Hz components in ASSR of the mixed-width response to the 4 Hz component in each of the fixed-width responses. In these contrasts plotted in Figure 8, a large area of the ROI showed significantly greater activity for the mixed-width condition compared to the fixed-width 16 ms condition; p = 0.05 threshold is *t = 3.08*; max t-value is *t = 4.40*, at the MNI co-ordinate 64, −20, 4; no voxels have negative t-values. Only one voxel showed significantly greater activity for the fixed-width 24 ms contrast; p = 0.05 threshold is *t = 3.13*, max t-value is *t = 3.13*, at the MNI co-ordinate 68, −26, −22; min t-value is *t = −0.87*. There were no voxels significant for the fixed-width 32 ms contrast; p = 0.05 threshold is *t = 3.26*; max t-value is *t = 2.26*; min t-value *is t = −1.1*.

## Discussion

This study tested whether variability in waveform shape affects the amplitude of the ASSR. This was done by presenting three cosinusoidal pulsed amplitude modulations as stimuli which either have a repetitive waveform shape, or have a waveform that varies pseudo-randomly between different widths of cosinusoidal pulsed AM. The principal finding is that when variability is introduced to stimuli that have a fixed modulation rate, the average responses to the same individual AM pulses are altered. A key factor in the design of the paradigm is that the spectra of the variable mixed-width AM stimuli, and the average spectra of the fixed-width AM stimuli, are identical. Hence, the assumption that there is a direct linear relationship between the spectra of the stimuli and the spectra of the responses is flawed. The specific null hypothesis; that there will be no significant difference between the ASSR to the mixed-width AM stimuli, and the average ASSR to the three fixed-width AM stimuli, is therefore rejected.

The beamforming contrasts in this study were performed within a defined ROI, the selection of which was based on the most consistent locus of activity in a previous study by Prendergast *et al.* [18]. Before we performed the experimental contrast between the mixed-width and pooled fixed-width responses, we verified the suitability of the ROI. Firstly, at the seed location, MNI 70, −26, −2, we calculated the grouped average spectra in virtual electrodes from the 18 participants. These spectra showed clear peaks at 4 Hz, and when the 4 Hz component in the responses is plotted against the 4 Hz energy in the respective AM stimuli, we see that the relationship is non-linear, see Figure 3. Notably, at this location, the relative amplitude of the fixed-width responses is similar to the Prendergast *et al.* [18] study, with the fixed width 24 ms giving the greatest response, the fixed-width 32 ms is the next largest, and the fixed-width 16 ms is the smallest response. In the beamformer contrasts, which are based on the amplitude of the 4 Hz component in the response spectra, we compare the 4 Hz activity in the ASSRs to the four AM conditions with the 4 Hz activity in response to a pure-tone. These contrasts all generate peaks of activity within the ROI that are in close proximity to the seed location. Hence this study which contrasts the amplitude of the response spectra generates similar peak loci to those in the Prendergast *et al.* [18], which uses both the amplitude and phase of the spectra with a T2 statistic. We are therefore confident that these analyses both verify the selection of the ROI, and also implicitly confirm that our beamformer methods are appropriate.

The main group-level ROI beamformer analysis demonstrates that within the defined ROI there is a significantly greater 4 Hz component to the mixed-width AM, than to the pooled fixed-width AM. This greater response to the mixed-width AM stimuli has a locus near the medial boundary of the STG and MTG, with a peak at the MNI coordinate 46, −26, −2. This significant difference at the group level was consistent with the trend observed at the individual level. In the analysis of the individual z-maps, 16 of the 18 participants showed some areas in the ROI that gave a greater response to the mixed-width AM stimuli, and other areas that gave a greater response to the fixed-width AM stimuli. However, across the group, there was selectivity to AM that was presented as mixed-width stimuli. The average maximum z-value for the positive z-maps was greater than the average minimum z-value for the negative z-maps, Figure 5, and at the group-level the largest positive and negative t-values are *t = 3.32* and *t = −0.2*, Figure 6.

Analysis of the grouped average spectra in virtual electrodes generated for each of the mixed-width and fixed-width conditions, at the MNI coordinate 46, −26, −2, confirm that we are observing an ASSR at this peak location rather than spurious non-phase locked activity, Figure 7 (left plot). Moreover, when the 4 Hz component in the responses is plotted against the energy at 4 Hz in the respective AM stimulus waveforms, Figure 7 (right plot), we again see a non-linear relationship, and we also observe that the responses to all the fixed-width stimuli are less than the response to the mixed-width stimuli. At this location, the mean normalised amplitude across the fixed-width responses is 0.89 of the amplitude in the mixed-width response. Interestingly, although the fixed-width 16 ms response has the smallest average amplitude, it is the only response that is relatively larger than what would be predicted from the energy in the response waveform. Hence, this further demonstrates the non-linearity of the fixed-width responses. However, the main finding from these figures is that the responses to the variable mixed-width stimuli have a greater average 4 Hz component than the fixed-width stimuli, even when the fixed-width stimuli have an equal or greater amount of 4 Hz energy in the stimulus waveform.

To further understand the relationship between the responses to the mixed-width stimuli, and to each of the fixed-width stimuli, we performed a final set of beamformer contrasts on the 4 Hz components in four respective ASSRs. These contrasts show that the responses to the mixed-width stimuli are significantly greater than the responses to the fixed-width 16 ms stimuli, however there is little or no significant difference with respect to the fixed-width 24 ms and fixed-with 32 ms responses. The observation that the significantly smaller response amplitude to the fixed-width 16 ms responses in not mirrored by a significantly larger response to the fixed-width 32 ms responses is further evidence for the non-linear relationship between the modulation spectrum and the response waveform.

The principal finding of this study is that adding variability in to the stimulus waveform generates a greater steady-state response than stimuli which have an equivalent mean energy at the stimulus modulation rate, but a waveform shape that is repetitive. We replicate the findings from Prendergast *et al.* [18], that the relationship between the spectra of the AM stimuli and the ASSR is non-linear when the spectra of the AM stimuli is varied at a fixed modulation rate. However, we also find that this relationship is non-linear when the spectra of the AM stimuli are matched, but one set of stimuli has variability in the waveform shape, and the other does not.

A dissociation between the modulation spectrum of an AM stimulus and behavioural discrimination thresholds is a well known phenomena in psychoacoustics. If the AM used is triangular rather than sinusoidal, then by using an asymmetric triangular modulation and time-reversing it, so called ‘ramped’ and ‘damped’ AM can be generated; which have different rates of onset of modulation, but identical AM spectra. These ‘ramped’ and ‘damped’ AM are easily discriminated [15]–[16], and the discrimination of ramped AM can be predicted from the change in the slope of an onset ramp, relative to the modulation cycle and independent of modulation rate [17]. Hence, there is strong perceptual evidence that modulation envelope processing is dependent on the shape of a modulation envelope, and independent of modulation rate; which is analogous to what we observe in this study.

Whilst this study may appear to be consistent with a model of modulation processing based on modulation waveform shape, rather than the modulation spectrum of the stimulus, the most parsimonious explanation for the relatively greater response to the variable mixed-width stimuli may be due to adaptation in the fixed-width ASSR. Psychoacoustically, modulation detection thresholds to sinusoidal AM are known to be affected by pre-exposure to both sinusoidal and non-sinusoidal AM stimuli [10]–[13], [31]. Green & Kay [11] also demonstrate, using sinusoidal, triangular and square wave AM adaptors, that the degree of adaptation is also dependent on the shape of the adapting waveform.

Adaptation to AM stimuli is also seen neurophysiologically. Bartlett & Wang [14] studied AM adaptation in the auditory cortex of marmoset monkeys and found that the spiking of neurones in response to sinusoidal AM stimuli could be both suppressed and facilitated by pre-exposure to another sinusoidal AM stimuli. The observed suppression was tuned to modulation frequency, and they note that the suppression was not solely based upon spectral properties of the stimuli, but was sensitive in particular to the temporal characteristics of preceding stimuli. They also note that the pattern of suppression was not related to spiking habituation.

An alternative explanation to for the greater response to the mixed-width stimuli may come from studies of ordered and disordered tone-pips. Chait *et al.* [32] studied the transition from either constant or regularly alternating tones, to a random sequence of tone-pips which alternate in frequency. The study found that there was an extra component in the average MEG response at the transition from the constant or regularly alternating tones to the random tone, with respect to what is observed at the transition from random tones to constant or regularly alternating tones. The inference from Chait *et al.* [32] is that in this study there may be extra components in the MEG response to the mixed-width AM stimuli. However, there is little evidence for such an interpretation, as this study specifically looks at the response at the modulation rate, and also, when we look at the averaged waveform to the mixed-width, and fixed-width AM stimuli, no extra component in the averaged waveform is observed.

With respect to the locus in the ROI at which we find the significant difference in ASSR between the mixed-width and fixed-width AM, it is at the medial boundary of the STG and MTG, at the MNI coordinate 46, −26. −2. However, the most consistent responses to cosinusoidal modulation pulse widths, as observed by Prendergast *et al.* [18] was at the MNI coordinate 70, −26, −2; and when we compare the 4 Hz component of the ASSR to each of the four AM stimuli in this study, with the 4 Hz component of the response to a pure tone, the peak response locations were similar to Prendergast *et al.* [18]. Hence, the location at which we observe the significantly greater ASSR to the mixed-width stimuli is different to the location of the greatest response to each of the respective cosinusoidal amplitude modulations.

The contrasts between each of the four AM stimuli and a pure tone, in Figure 4, suggest that whilst the peaks to the three fixed-width AM stimuli are relatively focal, the peak to the mixed-width AM stimuli is less focal, and more disparate. Hence, in the group-level contrast between the mixed width ASSR and the pooled fixed width ASSR, where there is a greater response to the mixed-width AM stimuli at the medial boundary of the STG and MTG, the greater response may be explained by the mixed-width AM stimuli stimulating a greater area of cortex. Alternatively, it may be that these medial and lateral loci have different functional roles, with the medial loci being selective for waveform shape.

There are functional consistencies in the temporal processing literature with the locus of the peak in the ROI at which we see the significant differences between the ASSRs to the mixed-width and fixed-width conditions. Boemio *et al.* [33] using fMRI to study the spectro-temporal properties of auditory processing, observe that both STG are sensitive to the local temporal structure of a stimulus, but the right hemisphere Superior Temporal Sulcus STS shows selectivity for slow temporal cues, of the order 200–300 ms, and the left hemisphere STS selectivity for rapid temporal cues in the order of 25–30 ms. Consistent with this is the Abrams *et al.* [34] evoked potential study which finds slow temporal features of speech (3–5 Hz) lateralizing to the right hemisphere, and rapid temporal feature of speech (20–50 Hz) lateralizing to the left hemisphere. We caution making too close a comparison between the Boemio *et al.* [33] and Abrams *et al.* [34] studies and this study, as this study used monaural presentation of the stimuli, to the left ear only.

In this study, the same component modulation stimuli, cosinusoidal AM pulses with half-width durations of 16 ms, 24 ms and 32 ms, were presented either as repetitive stimuli; the fixed-width AM stimuli, or pseudo-randomly; the mixed-width AM stimuli (see Figure 1). This internally-controlled presentation of the same pulsed AM stimuli generated a significantly greater response when the stimuli were presented in a pseudo-random order. Hence, by using pulsed AM, rather than continuous sinusoidal AM, and by adding variability into the presentation of the AM, we are able to observe changes in the ASSR. Therefore, whilst one-component sinusoidal modulations of auditory and visual cues are highly desirable for their simplicity, we feel it is important to acknowledge, as Georgeson *et al.* [35] observed in vision, that to understand the complexity of neural processing in brain, we may need to use complex stimuli, and look for non-linearities in sensory processing mechanisms.

### Conclusion

We find that stimulus variability does affect the amplitude of the auditory steady-state response. We therefore reject our null hypothesis, as we find that the ASSR to the mixed-width AM stimuli is greater than the average ASSR to the fixed-width AM stimuli. This finding is consistent with previous studies of AM adaptation, and suggests that analysis of waveform shape is a key feature of acoustic processing. The location at which we find the greater response to the mixed-width AM stimuli is different to the location where we find the greatest response to periodicity.
